# Nothing new under the sun

**DOI:** 10.1192/bjb.2022.50

**Published:** 2022-10

**Authors:** Paul Foster

**Affiliations:** Consultant psychiatrist in private practice with Psychiatric and Psychological Consultant Services (PPCS), London, UK. Email: dr.foster@sky.com.

I read with interest the editorial on illness narratives.^1^ But does this article say anything new regarding the importance of listening to the patient's story or offer any new insight into how to do this?

The understanding that it is much more important to know what sort of patient has a disease than what sort of disease a patient has is at least as old as William Osler (1849–1919) and probably dates to Hippocrates.

The authors consider it astonishing that half of care plans in the Care Programme Approach analysis had no evidence of recording patients’ views and one-third made no reference to carers. Might explanations for this be that there were no attempts to engage patients or carers who may not have been willing or able to be engaged or that the bureaucratic pressure and time involved in the recording process were experienced as barriers?
